# Bibliometric analysis of scientific publications in rheumatology journals from China and other top-ranking countries between 2007 and 2017

**DOI:** 10.7717/peerj.6825

**Published:** 2019-04-25

**Authors:** Chen Zhang, Xinzhe Feng, Chen Wang, Denghui Liu, Chongru He, Weidong Xu

**Affiliations:** 1 Department of Orthopedics, Changhai Hospital, Second Military Medical University, Shanghai, China; 2 Department of Traumatic Orthopedics, No. 906 Hospital, Ningbo, China

**Keywords:** Rheumatology, Bibliometrics, Science citation index expanded

## Abstract

**Objectives:**

Rheumatology-related diseases remain a significant burden worldwide. However, little is known about the comparative status of rheumatology research between Mainland China (MC) and the world’s leading countries. The aim of this study is to compare the quantity and quality of research output in the field of rheumatology that were written by researchers from MC, the USA, the UK, the Netherlands and France.

**Methods:**

Between 2007 and 2017, all articles published in 30 rheumatology journals were identified via Science Citation Index Expanded database. The number of total and annual articles, article types (randomized controlled trials (RCTs), reviews, case reports, clinical trials and meta-analysis), impact factor (IF), citations, h-index and articles in the high-impact journals were collected for quantity and quality comparisons. The correlation of socioeconomic factors and annual publications was also analyzed.

**Results:**

From 2007 to 2017, there were 53,439 articles published in rheumatology journals, of which researchers from the USA published 13,391 articles, followed by the UK, the Netherlands, France and MC with 6,179, 4,310, 4,066 and 2,898 articles, respectively. Publications from MC represented the ninth, but the number is growing rapidly. For total and average citations, MC still lags behind the other four countries in the study. Similar trends were observed in average IF, h-index and articles in the high-impact journals. In terms of article types, the USA occupies the dominant place, except for meta-analysis. The annual numbers of articles from MC and the USA were positively correlated with gross domestic product (*p* < 0.05).

**Conclusions:**

The USA has played predominant role in rheumatology research for the last 11 years. The annual number of published articles from MC has increased notably from 2007 to 2017. Although MC has made progress in the number of published articles over the past decade, it still lags far behind the highly developed countries in most bibliometric indicators. Thus, the general quality of publications from MC needs further improvement.

## Introduction

Rheumatic disorders are becoming one of the central health-care problems worldwide. Chronic rheumatic diseases are leading causes of disability, and bring heavy burden to the families and society (Xiang & Dai, 2009). By 2015, of the five main causes of global disability, two were related to rheumatism. (GBD 2013 DALYs and HALE Collaborators et al., 2015). The overall world prevalence of rheumatoid arthritis is approximately 0.5% to 1%. The incidence appears to be highest in Indians (5.3%) and lowest in people from China and Japan (0.2%–0.3%).

Rheumatology continues to be an exciting and vibrant specialty for specialists around the world. There have been important progress on the research front in rheumatology over the past 10 years (McQueen, 2017). In China, although the study of rheumatology started late, it is flourishing due to rapid development in the economy (Yamano & Nishioka, 2010). Numerous studies of rheumatic disorders have been published by Chinese researchers in recent years. In the past 10 years, more than US$26 million from Chinese government and private funding was granted to the study of rheumatology (Zeng et al., 2008). The quantity and quality of the scientific papers can reflect not only the level of individual research, but also the comprehensive national strength of a country. However, compared with other first-class countries, the research status of Chinese rheumatology is poorly understood. Bibliometrics is an important tool to analyze the literature of a certain scientific domain, and to assess the trends in research activity over time (Pu, Lyu & Su, 2016).

The aim of the study was to systematically evaluate rheumatology research from MC and the other four top-ranked countries between 2007 and 2017, and to provide a new perspective for future research directions. We investigated number and citations of publications, publication types, impact factors, and the relationship between gross domestic product (GDP) and the output of rheumatology research.

## Methods

### Search Strategy

In the present study, the Science Citation Index Expanded (SCIE) was used to perform the literature retrieval on September 5, 2018. A total of 30 journals related to rheumatology were included. Since the name of *Arthritis Rheum* journal was changed to *Arthritis Rheumatol* in 2014, articles published in journals using these two names were combined for the study. The details of the 30 journals were listed in Table S1. The ISSN numbers of the journals were used to perform the search. The final search query was “0003-4967 OR 1759-4790 OR 2326-5191 OR 0004-3591 OR 1462-0324 OR 1063-4584 OR 0049-0172 OR 1040-8711 OR 1478-6354 OR 1521-6942 OR 0889-857X OR 1297-319X OR 2151-464X OR 0315-162X OR 1523-3774 OR 0300-9742 OR 0392-856X OR 1756-1841 OR 0961-2033 OR 0770-3198 OR 1546-0096 OR 0172-8172 OR 1439-7595 OR 1471-2474 OR 1076-1608 OR 0482-5004 OR 0303-464X OR 0340-1855 OR 1309-0291 OR 1058-2452 OR 0341-051X” AND “USA[AD]”, “UK[AD]”, “Netherlands[AD]”, “France[AD]” and “China[AD] NOT Taiwan[AD] NOT Hong Kong[AD]” and “Language=English”. Data retrieval process was completed independently by two researchers, and any differences were resolved at the consensus meeting. The number of articles in clinical trial, review, case report, randomized controlled trial (RCT) and meta-analysis was obtained by searching PubMed.

### Data analysis

Four methods were used to compare scientific output in the five countries. First, the impact factors (IFs), h-index and citations were collected from Web of Science. The cumulative IF for a country over a year is simply the sum over all papers that the authors has published of the IFs of the journals. Country X has published three papers in journal A that has IF 1.2. It has published 1 papers in journal B that has IF 1.9. The cumulative IF would then be 5.5 (3*1.2+1*1.9). Second, publication types of the articles were calculated from Pubmed. Third, the 10 most published journals of rheumatology for each country and the number of articles published in the 10 most influential journals in each country were also counted. Finally, the h-index for each country were calculated. Statistical analyses were conducted using GraphPad Prism 6.0 (San Diego, CA, USA). Pearson’s correlation coefficient was used to study the relationship between the number of publications in different countries and GDP. The value of *p* < 0.05 was considered significant.

## Results

### Total amount and share of publications

**Figure 1:**
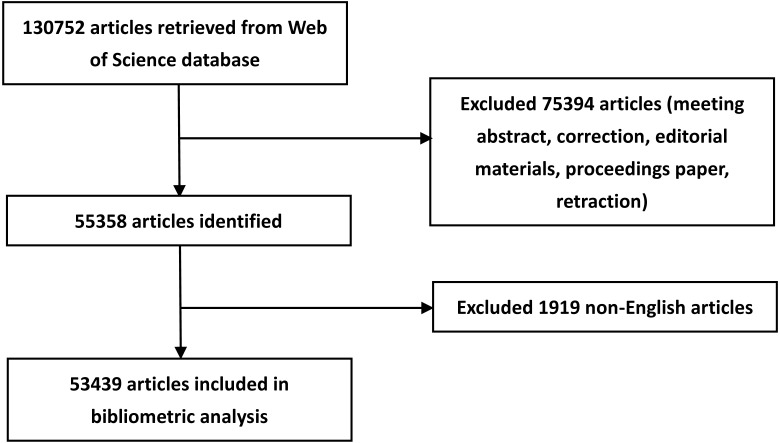
Flow chart of rheumatology research inclusion.

**Figure 2:**
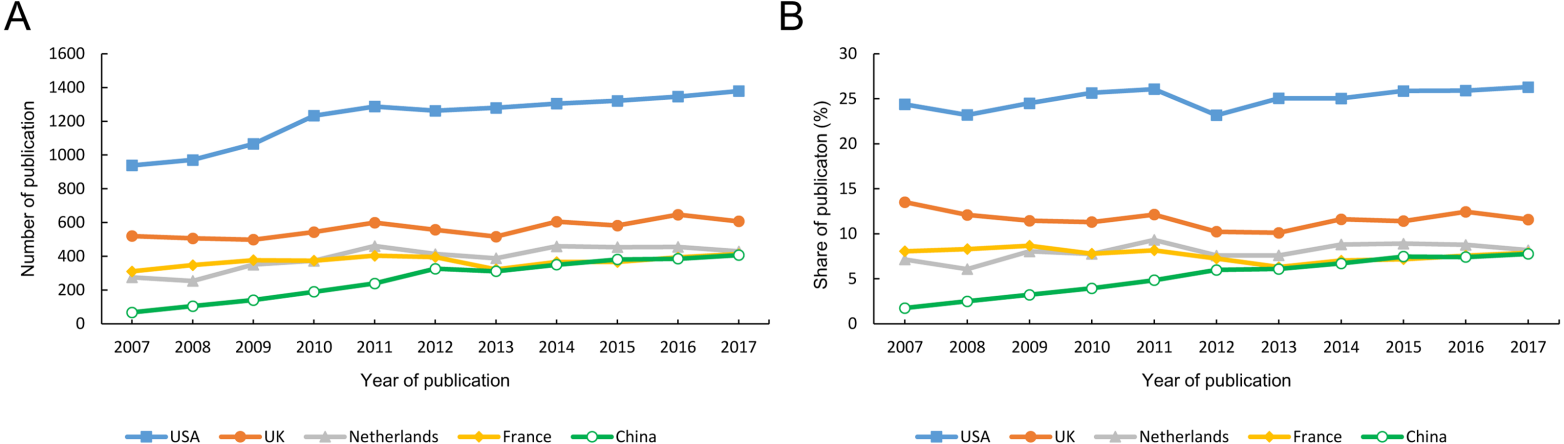
The number (A) and share (B) of papers published in rheumatology journals from the five countries.

A total of 53,439 articles (Fig. 1) were published between 2007 and 2017 in the 30 rheumatology journals. In general, the USA accounted for the largest proportion (13,391, 25.1%, 1st), followed by the UK (6,179, 11.6%, 2nd), the Netherlands (4,310, 8.1%, 3rd) and France (4,066, 7.6%, 4th). MC ranked 9th (2,898, 5.4%). We observed a significant increase in the number of articles published annually from the USA and MC since 2007, but those from the other three countries remained stable (Fig. 2A). Since 2015, the number of articles published in MC per year has exceeded that of France. The proportion of articles from MC has grown rapidly over time, but this was not the case for articles from the other four countries (Fig. 2B). Despite the growing number of publications, the share of articles from the USA remained stable for the last 11 years. From 2015 onwards, MC’s annual share of articles has surpassed that of France and approached that of the Netherlands. Among the five countries, the USA contributed the most number of top-cited articles (63).

### Publication activity in relation to GDP or number of authors

**Figure 3:**
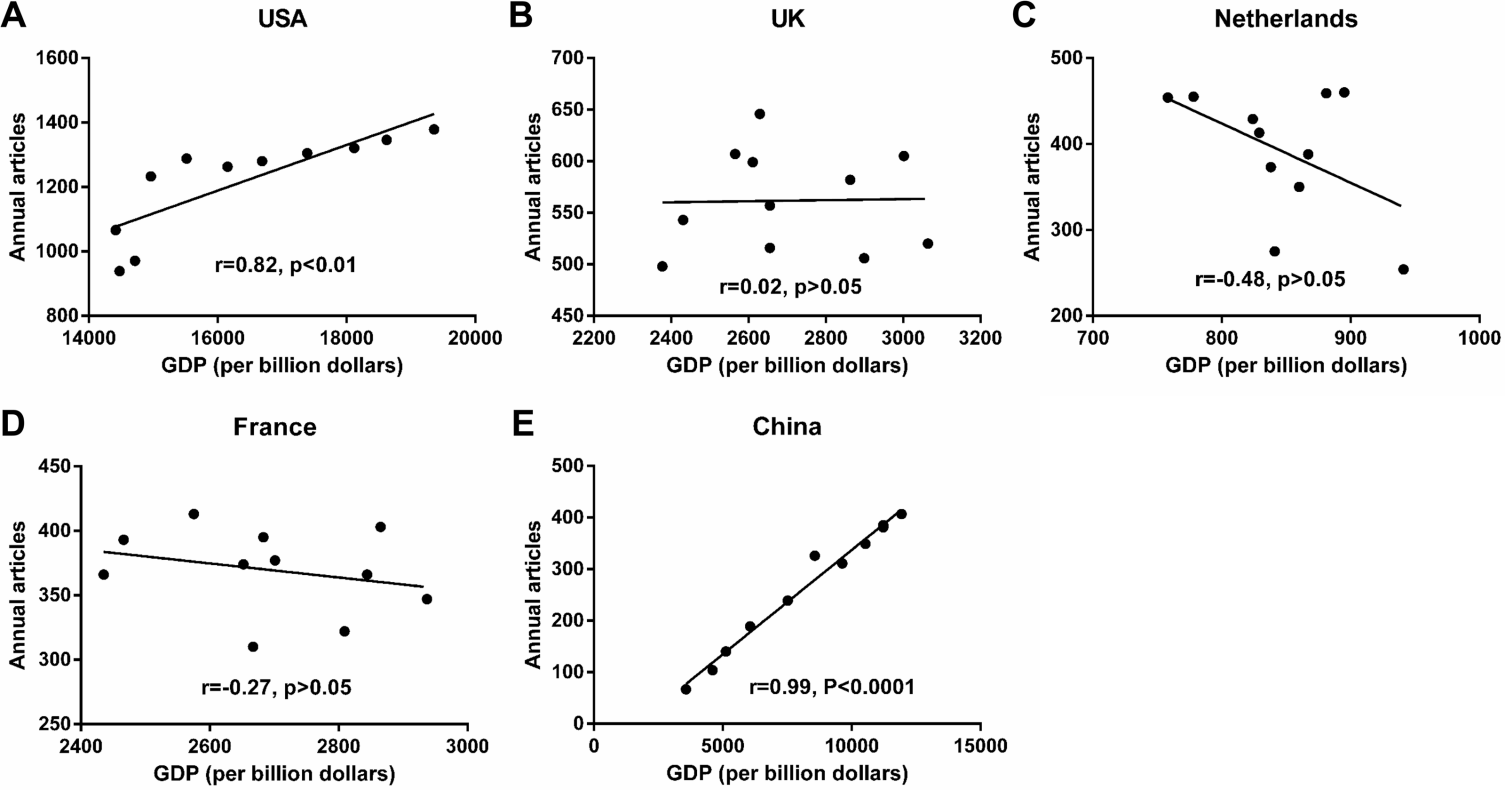
Influence of GDP on rheumatology publications by researchers from the USA (A), the UK (B), the Netherlands (C), France (D) and China (E) from 2007 to 2017. Abbreviations: GDP, gross domestic product.

**Figure 4:**
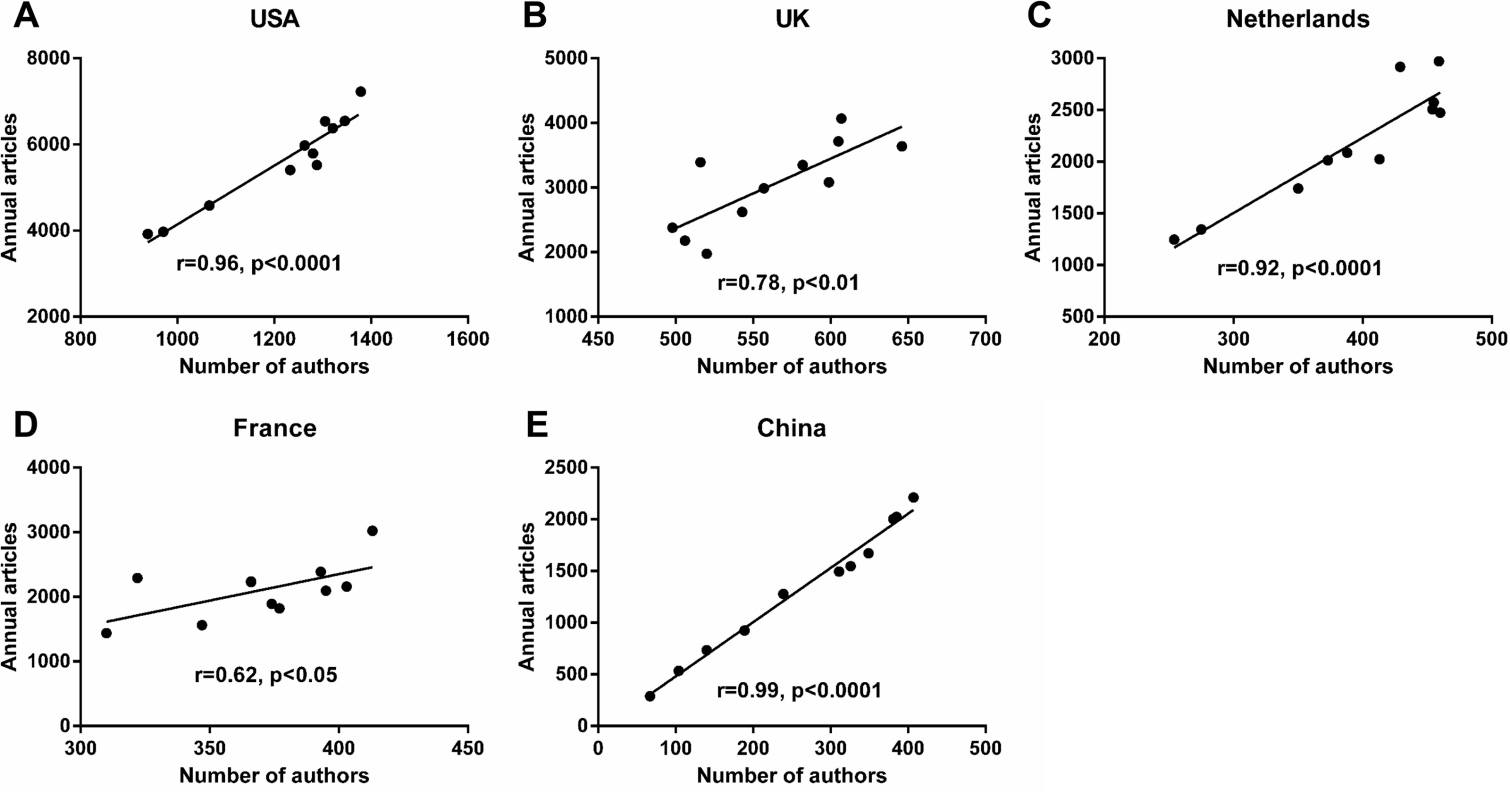
Influence of number of authors on rheumatology publications from the USA (A), the UK (B), the Netherlands (C), France (D) and China (E) from 2007 to 2017.

As illustrated in Fig. 3, annual articles in the USA (*r* = 0.82, *p* < 0.01) and MC (*r* = 0.99, *p* < 0.001) were strongly correlated with GDP. However, the annual number of articles published in the UK, the Netherlands and France was irrelevant to GDP. The number of authors from the five countries were listed in Table S2. We have observed that the number of authors in the five countries has continued to increase since 2007. And annual articles in the five countries were strongly correlated with their annual number of authors (Fig. 4).

### Publication types

**Figure 5:**
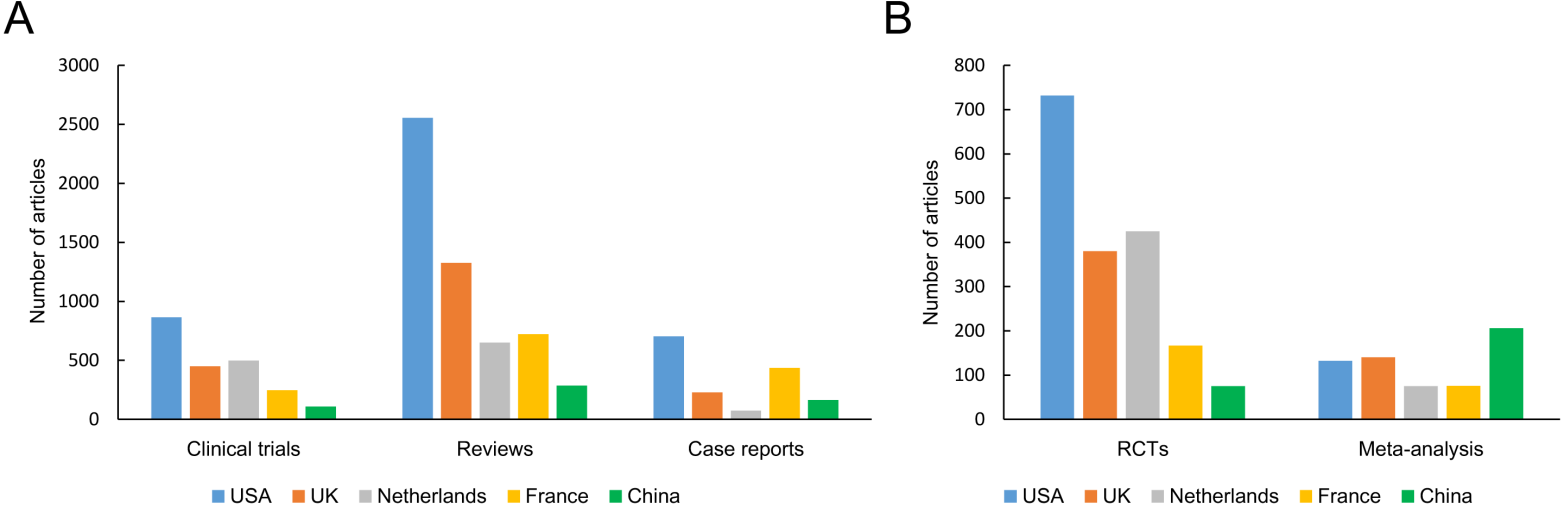
The number of papers of each different publication type (including RCTs, clinical trials, reviews, case reports and meta-analysis) from different countries. Abbreviations: RCT, randomized control trial.

**Figure 6:**
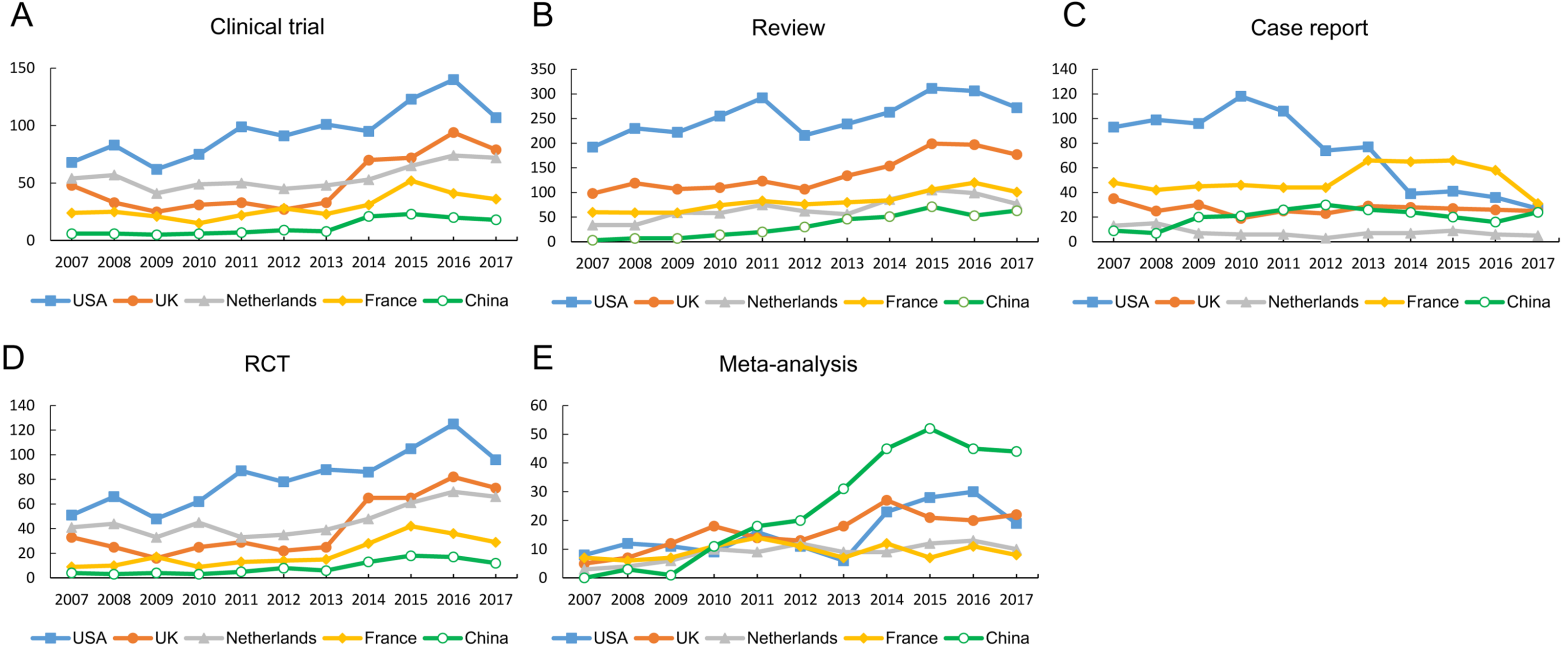
The annual number of five article types published by the five countries.

The number of 5 types of articles published by these countries, including clinical trial, RCT, case report, review and meta-analysis, was shown in Fig. 5. The USA accounted for the largest proportion of all types of articles except meta-analysis. In respect of RCTs and clinical trials, the total numbers from MC were remarkably lower than those from the other countries. MC has published the largest number of meta-analyses in the five countries over the past 11 years. The annual number of five article types published by five countries were shown in Fig. 6. Since 2014, the annual number of RCTs and clinical trials published in the USA has increased significantly. However, the annual number of case reports showed an opposite downward trend since 2010. Except for the increase in the number of meta-analysis and reviews since 2009, there have been no significant changes in the other four types of articles published in MC.

### Impact factors

According to JCR 2017, all the 30 journals had IFs. The details of the accumulated and average IFs from the five countries were listed in Table 1. According to the accumulated IF calculation, MC (10,201) was lower than the USA (65,493), the UK (34,606), the Netherlands (26,844) and France (22,653) in the past 11 years. The total average IFs were arranged in the following order: the Netherlands (6.2), the UK (5.6), France (5.6), the USA (4.9), and MC (3.5). In addition, the average IF of MC decreased year by year. And MC’s average IFs were negatively correlated with the annual number of articles (*r* = 0.86, *p* < 0.001).

**Table 1:** Cumulative and average IFs for articles from the five countries.

	Cumulative IF					Average IF				
Year	USA	UK	Netherlands	France	MC	USA	UK	Netherlands	France	MC
2007	4,593	2,807	1,844	1,350	256	5.5	6.2	7.8	7.0	4.7
2008	4,934	2,813	1,652	1,743	397	5.4	6.9	6.4	7.3	4.6
2009	5,163	2,802	2,337	1,977	482	5.4	7.0	8.7	8.4	3.4
2010	6,237	3,232	2,481	2,291	684	6.0	7.3	8.4	8.1	3.8
2011	6,245	3,460	2,944	2,351	961	5.9	7.7	8.4	9.3	3.8
2012	6,166	3,065	2,577	2,250	1,066	6.3	7.4	8.1	8.1	3.6
2013	6,153	2,775	2,398	2,006	957	6.6	5.6	6.4	7.2	3.2
2014	6,443	3,413	2,741	2,198	1,318	6.7	4.9	5.7	6.0	3.3
2015	6,454	3,323	2,732	1,995	1,428	5.5	4.1	5.0	4.3	3.2
2016	6,563	3,544	2,618	2,285	1,421	5.3	4.1	4.6	4.4	3.2
2017	6,542	3,372	2,520	2,207	1,231	4.7	5.6	5.9	5.3	3.0
Total	65,493	34,606	26,844	22,653	10,201	4.9	5.6	6.2	5.6	3.5

**Notes.**

IF impact factor MC Mainland China

### Citation reports and H-index

**Figure 7:**
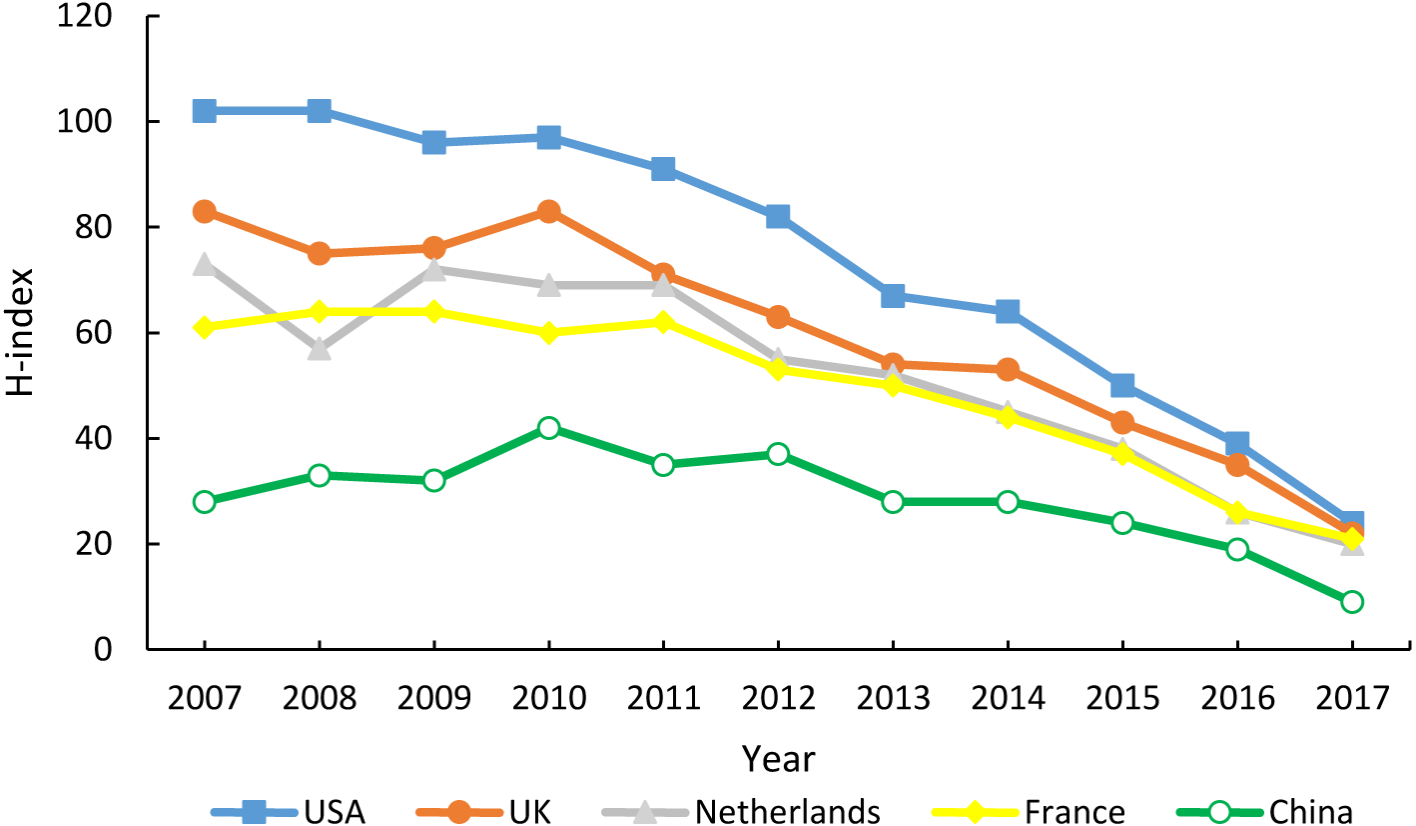
The h-index of the five countries.

The details of the total and average citations from the five countries were listed in Table 2. The USA had the largest number of total citations and the Netherlands had the most average citations in the past 11 years, while MC’s total and average citations were the lowest. Total citations from the five countries have increased from 2007 to 2010. But after that, the number of citations have declined year by year. As shown in Fig. 7, the USA had the highest value of h-index (185), followed by the UK (154), the Netherlands (140), France (132) and MC (72).

**Table 2:** Total and average citations of articles from the five countries.

Year	USA	UK	Netherlands	France	MC
2007	43,290	24,312	16,271	12,752	2,081
2008	46,372	22,163	11,788	14,367	3,220
2009	45,199	23,610	19,504	16,607	5,507
2010	53,755	30,496	21,830	18,762	5,400
2011	44,716	21,942	18,311	14,707	5,166
2012	41,054	17,728	12,145	12,215	6,114
2013	31,212	15,332	12,507	10,514	4,020
2014	26,894	15,382	11,232	9,587	3,955
2015	18,844	8,939	6,601	5,374	3,348
2016	12,489	6,800	4,536	3,805	1,994
2017	5,509	3,469	2,367	2,332	905
Total citations	369,334	190,173	137,092	121,022	41,710
Average citations	27.6	30.8	31.8	29.8	14.4

**Notes.**

MC Mainland China

### Top 10 high-impact rheumatology journals

In the past 11 years, the five countries have published 18,310 articles in the top 10 rheumatology journals. We found that 56.7% (10,380/18,310) of the articles were published in the top four journals, including *Nat Rev Rheumatol, Ann Rheum Dis*, *Arthritis Rheumatol* and *Osteoarthr Cartilage*. The USA published the most articles (8,389, 45.8%) in the top 10 rheumatology journals, followed by the UK (4,061, 22.2%), the Netherlands (2,916, 15.9%), France (2,056, 11.2%) and MC (888, 4.8%). As shown in Table 3, 62.7% of the US articles were published in the 10 top-ranking journals, while only 30.6% of the articles in MC were published in these journals.

**Table 3:** Articles in the top 10 high-impact rheumatology journals from the five countries.

Rank	Journal title	2017 IF	USA	UK	Netherlands	France	MC
1	*NAT REV RHEUMATOL*	15.661	228	108	69	31	19
2	*ANN RHEUM DIS*	12.350	1,200	1,036	1,049	640	131
3	*ARTHRITIS RHEUMATOL*	7.871	2,186	666	499	419	192
4	*OSTEOARTHR CARTILAGE*	5.454	1,043	308	254	130	172
5	*RHEUMATOLOGY*	5.245	624	1,126	404	353	124
6	*SEMIN ARTHRITIS RHEU*	4.356	311	67	40	91	22
7	*CURR OPIN RHEUMATOL*	4.277	499	110	59	37	9
8	*ARTHRITIS RES THER*	4.269	850	348	331	203	189
9	*ARTHRIT CARE RES*	4.149	1,084	254	198	141	29
10	*RHEUM DIS CLIN N AM*	3.522	364	38	13	11	1
Total			8,389	4,061	2,916	2,056	888
Divided by total article volume of each country (%)			62.7	65.7	67.7	50.6	30.6

**Notes.**

IF impact factor MC Mainland China

### Most published rheumatology journals

The journals with the most published articles by researchers in the five countries were shown in Table 4. Seven of the 10 most published journals by the US researchers were among the top 10 influential journals. Meanwhile, six journals in the UK, six journals in the Netherlands and seven journals in France were among the top 10 high IF journals. Only three journals in MC were among the top 10. Over the past 11 years, *Bmc Musculoskel Dis* ranked the first in MC. However, it was not ranked top 10.

**Table 4:** Top 10 most published rheumatology journals in the five countries.

	USA	N	UK	N	Netherlands	N	France	N	MC	N
1	*AR*	2,186	*R*	1,126	*ARD*	1,049	*JBS*	982	*BMD*	374
2	*JOR*	1,429	*ARD*	1,036	*AR*	499	*ARD*	640	*RI*	360
3	*ARD*	1,200	*AR*	666	*R*	404	AR	419	*CR*	332
4	*ACR*	1,084	*JOR*	494	*JOR*	370	*JOR*	387	*LUPUS*	200
5	*OAC*	1,043	*BMD*	393	*BMD*	335	*R*	353	*ART*	189
6	*ART*	850	*ART*	348	*ART*	331	*CAER*	224	*JOR*	179
7	*R*	624	*OAC*	308	*OAC*	254	*ART*	203	*OAC*	172
8	*JCR*	530	*ACR*	254	*ACR*	198	*ACR*	141	*IJORD*	160
9	*LUPUS*	525	*CAER*	232	*CAER*	187	*OAC*	130	*ARD*	131
10	*COIR*	499	*CR*	230	*CR*	143	*SAR*	91	*CAER*	127

**Notes.**

*ARD* ANN RHEUM DIS, IF = 12.350 *AR* ARTHRITIS RHEUMATOL, IF = 7.871 *R* RHEUMATOLOGY, IF = 5.245 *OAC* OSTEOARTHR CARTILAGE, IF = 5.454 *COIR* CURR OPIN RHEUMATOL, IF = 4.277 *ART* ARTHRITIS RES THER, IF = 4.269 *ACR* ARTHRIT CARE RES, IF = 4.149 *JOR* J RHEUMATOL, IF = 3.470 *CAER* CLIN EXP RHEUMATOL, IF = 3.201 *IJORD* INT J RHEUM DIS, IF = 2.423 *LUPUS* LUPUS, IF = 2.969 *CR* CLIN RHEUMATOL, IF = 2.141 *RI* RHEUMATOL INT, IF = 1.952 *BMD* BMC MUSCULOSKEL DIS, IF = 1.998 *JCR* JCR-J CLIN RHEUMATOL, IF = 1.974 *JBS* JOINT BONE SPINE, IF = 3.304 *SAR* SEMIN ARTHRITIS RHEU, IF = 4.356 IF impact factor MC Mainland China

## Discussion

Rheumatism is a major cause of disability around the world. As far as we know, this is the first study to compare the contributions of authors from MC and the top countries in this field to rheumatology research. It is unrealistic to compare the publications of MC with that of all countries in the world. Therefore, we only selected the top four countries in the field of rheumatology for comparison, which have made great contributions to this field.

Our study compared the research level of rheumatology between MC and the USA, the UK, the Netherlands and France from 2007 to 2017. The results showed that American researchers published the most articles among the five countries. Although the annual number of articles from the USA increased year by year, the share of articles remained steady. Notably, the share and number of articles published each year in MC have increased dramatically. The number of articles published by MC in 2017 was more than 6 times in 2007 and was quite close to that of the Netherlands. Its rapid growth might due to the continuous increase in GDP and number of rheumatologists. In addition to the improvement in economic status, the increase in research and development funds was undoubtedly the main reason for the progress of China’s scientific output (Zeng et al., 2008). At the same time, more and more rheumatologists were engaged in rheumatology research. Other factors such as incentive reward plan and career needs would certainly stimulate research output (Man et al., 2014). However, considering the huge population size, MC’s research in the field of rheumatology lagged far behind other developed countries . However, it has to be mentioned that the correlation between GDP and publications should be carefully interpreted, as the GDP growths in other countries were much smaller than in the USA and MC, so the correlation will be weak.

Good RCTs were often considered the gold standard in testing the efficacy or effectiveness of medical intervention (Schulz et al., 2010). In the past 11 years, the USA published the largest number of RCTs, clinical trials, reviews and case reports. It is noteworthy that MC published the fewest number of RCTs and clinical trials among the five countries, indicating that a less quantity of original work is made available by MC. Clinical research has bridged the gap between basic science and human health improvement. It is heavily weighted towards biomedical science, and plays a special role in the fight against rheumatic diseases by providing evidence for their treatment and diagnosis. Based on the advantages of clinical research, more clinical studies should be performed to provide new insights into the prevention, biomarkers, diagnosis or treatment of rheumatic diseases.As is known to all, MC has the largest population in the world, thus accumulated a large number of clinical data. However, these resources have not been fully utilized. Controlled clinical studies should be an important research direction of Chinese rheumatologists in the future. High-quality clinical research is expensive, and in the future should receive more funding support. It should be mentioned that the number of RCTs and clinical trials were probably in relation with the development of pharmaceutic industry devoted to the discovery of original drugs because the industry not only designed but also financed and promoted the majority of those kinds of publications. MC’s research in this area is still far from that in developed countries. Therefore, new drug research or other original research should be important research directions of Chinese rheumatologists in the future. Our results also showed that the number of meta-analysis from MC was the most among the five countries. Though a meta-analysis is a secondary source, it combine the results from multiple studies in an effort to increase power, and improves estimates of the size of the effect or resolve uncertainty. It is worth mention that the number of RCTs in the USA was far ahead of the other four countries and has grown year by year since 2009. This might be the reason why the total citations in the USA still ranked first.

The IF is a measure of the frequency with which the average article in a journal has been cited in a particular year (Zou, Li & Xu, 2016). It is used to measure the importance of a journal by calculating the number of times it’s articles are cited (Cherubini, 2008). Although there are many deficiencies, IF is a good technique for scientific evaluation. The annual total IFs of articles originating in MC has increased significantly over the past 11 years, but was still markedly lower than those from the other four countries. Interestingly, the average IFs from MC showed a linear decrease during 2010–2017. Also, we found that MC’s average IFs were negatively correlated with its annual number of articles. This indicated that rheumatology researchers from MC may have overemphasized the quantity of articles, while ignoring the quality of articles (Li et al., 2010; Xu et al, 2011). It should be noted that the IF is not always a reliable instrument to measure the quality of articles (Wang, Song & Barabasi, 2013). It is possible that articles published in low-impact journals could be excellent research, and vice versa. Therefore, we chose h-index and citations of articles for further comparison. Times cited of an article represents the extent to which it affects other publications (Jiang et al., 2016). This makes it easy to find some of the most important articles in a field. The h-index is a number intended to measure both the productivity and the citation impact of a scientist or scholar (Bornmann & Daniel, 2009). Although the number of articles published in MC has been growing rapidly each year, the number of citations per article was still the lowest among the five countries. The same results were found in the most popular rheumatology journals and the h-index. All these data points to the urgent need for MC to improve the quality of its publications.

There are some limitations in our study. First, only 30 rheumatology journals were included in the experiment. Many general journals also published rheumatology articles and were excluded from the study. Second, the IFs, citations and ranks of journals were calculated according to JCR 2017. But the data of JCR changes each year. Third, a bias may exist because we determined the country of a paper according to the first affiliation of the first author (Ye et al., 2014). The contributions of authors from other countries in some national cooperation projects may be overlooked. In addition, it is better to combine disease incidence with the publications. This will guide the monitoring of the disease.

## Conclusions

In summary, this study provided an overview of global rheumatology research for a decade. Although MC has made great progress in rheumatology research, the USA is still dominates. It should be emphasized that MC still has a long way to go to achieve the academic performance of the USA and the UK. Of note and worth mentioning is the fact that MC has published the lowest number of clinical trials and RCTs. As the second largest economy in the world with a population of over 1.3 billion, MC has great potential in the field of rheumatology. We suggest that MC researchers should spend less time writing meta-analysis and more time on all other types of publications (clinical studies, comparative studies, consensus, guidelines, basic and experimental research, epidemiologic studies, educational studies, etc.).

##  Supplemental Information

10.7717/peerj.6825/supp-1 Table S1 The list of the 30 rheumatology journals

10.7717/peerj.6825/supp-2 Table S2 Annual number of authors from the five countries

## Additional Information and Declarations

### Data Availability

The following information was supplied regarding data availability: The raw measurements are available in the Supplemental Files.
