# Supplementary material for: Bibliometric analysis of scientific publications in rheumatology journals from China and other top-ranking countries between 2007 and 2017
Source: PeerJ. 2019 Apr 25;7:e6825. doi: 10.7717/peerj.6825 (PMC6487180; doi:10.7717/peerj.6825)
Supplement: Table S1 [file peerj-07-6825-s001.docx]

|  | ISSN | Full Name | Abbreviated Journal Title | 2007 IF |
| --- | --- | --- | --- | --- |
| 1 | 0003-4967 | Annals of The Rheumatic Diseases | ANN RHEUM DIS | 12.35 |
| 2 | 1759-4790 | Nature Reviews Rheumatology | NAT REV RHEUMATOL | 15.661 |
| 3 | 2326-5191/0004-3591 | Arthritis & Rheumatology (Arthritis and rheumatism) | ARTHRITIS RHEUMATOL (ARTHRITIS RHEUM) | 7.871 |
| 4 | 1462-0324 | Rheumatology | RHEUMATOLOGY | 5.245 |
| 5 | 1063-4584 | Osteoarthritis And Cartilage | OSTEOARTHR CARTILAGE | 5.454 |
| 6 | 0049-0172 | Seminars In Arthritis And Rheumatism | SEMIN ARTHRITIS RHEU | 4.356 |
| 7 | 1040-8711 | Current Opinion In Rheumatology | CURR OPIN RHEUMATOL | 4.277 |
| 8 | 1478-6354 | Arthritis Research & Therapy | ARTHRITIS RES THER | 4.269 |
| 9 | 1521-6942 | Best Practice & Research In Clinical Rheumatology | BEST PRACT RES CL RH | 3.198 |
| 10 | 0889-857X | Rheumatic Disease Clinics of North America | RHEUM DIS CLIN N AM | 3.522 |
| 11 | 1297-319X | Joint Bone Spine | JOINT BONE SPINE | 3.304 |
| 12 | 2151-464X | Arthritis Care & Research | ARTHRIT CARE RES | 4.149 |
| 13 | 0315-162X | Journal of Rheumatology | J RHEUMATOL | 3.150 |
| 14 | 1523-3774 | Current Rheumatology Reports | CURR RHEUMATOL REP | 3.47 |
| 15 | 0300-9742 | Scandinavian Journal of Rheumatology | SCAND J RHEUMATOL | 3.021 |
| 16 | 0392-856X | Clinical And Experimental Rheumatology | CLIN EXP RHEUMATOL | 3.201 |
| 17 | 1756-1841 | International Journal of Rheumatic Diseases | INT J RHEUM DIS | 2.423 |
| 18 | 0961-2033 | Lupus | LUPUS | 2.969 |
| 19 | 0770-3198 | Clinical Rheumatology | CLIN RHEUMATOL | 2.141 |
| 20 | 1546-0096 | Pediatric Rheumatology | PEDIATR RHEUMATOL | 2.543 |
| 21 | 0172-8172 | Rheumatology International | RHEUMATOL INT | 1.952 |
| 22 | 1439-7595 | Modern Rheumatology | MOD RHEUMATOL | 1.955 |
| 23 | 1471-2474 | Bmc Musculoskeletal Disorders | BMC MUSCULOSKEL DIS | 1.998 |
| 24 | 1076-1608 | Jcr-Journal of Clinical Rheumatology | JCR-J CLIN RHEUMATOL | 1.974 |
| 25 | 0482-5004 | Revista Brasileira De Reumatologia | REV BRAS REUMATOL | 1.35 |
| 26 | 0303-464X | Acta Reumatologica Portuguesa | ACTA REUMATOL PORT | 1.105 |
| 27 | 0340-1855 | Zeitschrift Fur Rheumatologie | Z RHEUMATOL | 0.697 |
| 28 | 1309-0291 | Archives of Rheumatology | ARCH RHEUMATOL | 0.208 |
| 29 | 1058-2452 | Journal of Musculoskeletal Pain | J MUSCULOSKELET PAIN | 0 |
| 30 | 0341-051X | Aktuelle Rheumatologie | AKTUEL RHEUMATOL | 0.094 |

**Table S1 The list of the 30 rheumatology journals.**
