# Supplementary material for: Bibliometric analysis of scientific publications in rheumatology journals from China and other top-ranking countries between 2007 and 2017
Source: PeerJ. 2019 Apr 25;7:e6825. doi: 10.7717/peerj.6825 (PMC6487180; doi:10.7717/peerj.6825)
Supplement: Table S2 [file peerj-07-6825-s002.docx]

**Table S2 Annual number of authors from the five countries.**

| Year | USA | UK | Netherlands | France | MC |
| --- | --- | --- | --- | --- | --- |
| 2007 | 3923 | 1975 | 1345 | 1438 | 289 |
| 2008 | 3974 | 2179 | 1247 | 1563 | 534 |
| 2009 | 4582 | 2377 | 1740 | 1822 | 734 |
| 2010 | 5407 | 2623 | 2013 | 1890 | 924 |
| 2011 | 5523 | 3082 | 2473 | 2159 | 1279 |
| 2012 | 5978 | 2987 | 2024 | 2094 | 1547 |
| 2013 | 5794 | 3393 | 2086 | 2292 | 1494 |
| 2014 | 6535 | 3714 | 2971 | 2237 | 1672 |
| 2015 | 6375 | 3347 | 2507 | 2227 | 2004 |
| 2016 | 6547 | 3639 | 2574 | 2387 | 2024 |
| 2017 | 7227 | 4067 | 2917 | 3022 | 2212 |

**Notes.**

MC, Mainland China.
